# Ultra weak photon emission—a brief review

**DOI:** 10.3389/fphys.2024.1348915

**Published:** 2024-02-14

**Authors:** Rhys R. Mould, Alasdair M. Mackenzie, Ifigeneia Kalampouka, Alistair V. W. Nunn, E. Louise Thomas, Jimmy D. Bell, Stanley W. Botchway

**Affiliations:** ^1^ Research Centre for Optimal Health, School of Life Sciences, University of Westminster, London, United Kingdom; ^2^ OCTOPUS, Central Laser Facility, Science and Technology Facilities Council, Didcot, United Kingdom; ^3^ The Guy Foundation, Beaminster, United Kingdom

**Keywords:** biophoton, ultraweak photon emission, bystander effect, non-chemical signalling, radicals, biological autoluminescence

## Abstract

Cells emit light at ultra-low intensities: photons which are produced as by-products of cellular metabolism, distinct from other light emission processes such as delayed luminescence, bioluminescence, and chemiluminescence. The phenomenon is known by a large range of names, including, but not limited to, biophotons, biological autoluminescence, metabolic photon emission and ultraweak photon emission (UPE), the latter of which shall be used for the purposes of this review. It is worth noting that the photons when produced are neither ‘weak’ nor specifically biological in characteristics. Research of UPE has a long yet tattered past, historically hamstrung by a lack of technology sensitive enough to detect it. Today, as technology progresses rapidly, it is becoming easier to detect and image these photons, as well as to describe their function. In this brief review we will examine the history of UPE research, their proposed mechanism, possible biological role, the detection of the phenomenon, and the potential medical applications.

## 1 Introduction

### 1.1 History of ultraweak photon emission (UPE) research

The prevailing consensus in modern biochemistry has been that cells and their organelles communicate via a plethora of chemical means, including numerous molecular signal and receptor interactions, ion release/uptake exchanges, secondary messengers, and signal cascades. In the 1920s, the Russian scientist Alexander G. Gurwitsch explored the possibility of a novel non-chemical and non-contact method of cell-to-cell communication (Gurwitsch, 1923). In a series of experiments, he positioned the tip of an onion root (called the inducer) towards a second onion root (the receiver), separated by quartz. The receiver displayed an increased rate of mitosis compared to the control receiver, which was separated from the inducer by an opaque barrier. Gurwitsch proposed that the inducer was transmitting a non-chemical signal (possibly electromagnetic), through a process he termed “mitogenic radiation” (Gurwitsch, 1923; Gurwitsch and Gurwitsch, 1960).

In the 1930s, further evidence of mitogenic radiation was provided by several groups using modified Geiger Counters (Cifra et al., 2011). Gurwitsch’s work incited great interest at the time, yet by the 1940s-50s the field had somewhat faded into obscurity, with only a handful of scientists pursuing the phenomenon, exacerbated by a shift towards molecular biology research and World War 2 (Scholkmann et al., 2013). Indeed, most of these earlier studies were subject to similarly broad critiques, namely, a lack of adequate statistical analysis, failure to account for potential confounders such as volatile chemical communication, and perhaps most fundamentally, the lack of direct evidence for an electrochemical signal (and associated receptors) (Bateman, 1935), with Irving Langmuir going so far as to labelling Gurwitsch’s “mitogenic rays” as nothing more than “*pathological science, driven by subjectiveness and wishful thinking*” (Langmuir and Hall, 1989).

Despite such damning appraisals, study in the field continued, mainly at the fringes of biological research. In 1980, Kaznacheev and colleagues treated a range of cultured tissues with different viruses and chemical agents (Cifra et al., 2011). They observed that degradation of treated cultures was mirrored in adjacent, untreated cultures separated by quartz dividers of various thickness (Kaznacheev et al., 1980). In 1984, Bat’yanov performed comparable experiments using isolated rat liver mitochondria, showing that stimulation with succinate and adenosine diphosphate elicited changes in oxygen consumption in untreated mitochondria separated by quartz (Scholkmann et al., 2013). He concluded, like Gurwitsch and Kazancheev before him, that the interaction between isolated material must be non-chemical (photonic) in nature, specifically within the ultraviolet (UV) range (around 200–400 nm) (Bat’yanov, 1984). In 1984, the German biophysicist Fritz Albert-Popp coined the term “*biophoton*”, describing a photon originating from a biological system, of non-thermal origins in the UV-visible range (Popp et al., 1984). This phenomenon was said to be distinct from bioluminescence, which is based on the luciferin-luciferase enzymatic mechanism found in certain species (Fleiss and Sarkisyan, 2019). Popp proposed that “biophotons” were produced in what he described as a coherent field (where the subunits of the biological system act in a “cooperative manner”) and could theoretically regulate all types of cellular processes (Popp, 2003). Although his vision for biophotons is yet to be fully realised experimentally, Popp’s work has been hugely influential for re-starting research in the field of non-chemical communication in biological systems. However, Popp’s work did not differentiate between delayed luminescence and photons produced only from biological activity (see below). The detection of ultraweak photon emission (UPE) has now been reported originating from bacteria (Laager et al., 2009), fungi (Quickenden and Tilbury, 1991), seeds (Gallep and Dos Santos, 2007) and animal tissues (Cohen and Popp, 2003).

Other investigations of non-chemical cell communication have been reported, including the detection of growth orientation by cellular populations mediated by light in the red (around 620–750 nm) to infrared (IR) (around 750–1,000 nm) range, forming a “rudimentary form of cellular vision” (Albrecht-Buehler, 1992). Responses in isolated neutrophils have also been reported following induction of respiratory burst in isolated, adjacent cells (Shen et al., 1994). More recently, researchers have attempted to control for potential confounding influences by building bespoke experimental set ups; Farhadi et al. found that H_2_O_2_ treatment of intestinal epithelial cells induced a reduction in total protein content, an increase in nuclear factor kappa B (NFκβ) activation and structural damage in detector cells isolated from inducer cells using a custom-made container (Farhadi et al., 2007). Other set-ups, including a dish-in-dish design, demonstrated that apoptotic or cancerous cells can induce abnormal Ca^2+^ flux in neighbouring cells free from non-photonic external electromagnetic influence (Chaban et al., 2013). Furthermore, a “dish-on-dish” design found that endothelial cells altered the growth and morphology of cells placed below in a separate dish, an effect that disappeared once a black filter was used to prevent the passage of light between the two populations (Rossi et al., 2011).

Whilst the above research observes what could be described as “passive” UPE-based communication, several studies have also assessed the effects of “induced” UPE exposure on cellular biology. For example,. beta-irradiation of HaCaT cells induced a significant “secondary emission of photons” in the UV-A range, which in turn resulted in the cell death of neighbouring, non-irradiated cells (Le et al., 2015a), referred to as the bystander effect (Le et al., 2015b). However, this work is complicated in its approach in that the radiation source was still present during the UPE detection. There is also growing evidence that the wavelengths of UPE are integral to their biological effects; isolated populations of the unicellular protozoa *Paramecium caudatum* experienced contrasting changes in growth depending on whether they were mutually visible through >150 nm or >340 nm light (Fels, 2009). It is worth noting that exposure of most biomolecules, including DNA, to 150 nm light would induce one-photon photoionization leading to damage.

Despite these results, significant debate persists as to whether light-based influence provides a satisfactory explanation for the reported phenomena (Volodyaev and Beloussov, 2015). Other potentially conceivable mechanisms, including volatile and sound-based signalling, have also been proposed, although some of the bespoke experimental protocols described above have made these alternative explanations unlikely. Our own recent work indicated that volatile compound induced emission can lead to cell-to-cell communication and should not be ignored (Mould et al., 2022)., although we have also found evidence of non-chemical communication in isolated mitochondria in experiments with a greater degree of protection against potential volatile effects (Mould et al., 2023). However, there is a more significant conundrum associated with the potential of UPE as a non-chemical form of communication, mainly that they are of a very low intensity (rather than ultralow energy) suggesting that cells may not have the capacity to detect these signals above the cellular ambient “biological noise” (Scholkmann et al., 2013). Indeed, Cifra et al. submit that in order to transmit information via electromagnetic waves, cells must have the capability to *generate*, *detect* and *sense* specific properties of these waves, for which, they argue, there is little concrete evidence, concluding that light-based cellular signalling is either a paradox, or not naturally accomplishable (Kučera and Cifra, 2013).

On both sides of the debate, reviewers agree that the extremely low intensity of UPE is a key reason for the discipline’s challenging nature. One interesting possibility is that the intensity of UPE may be considerably higher inside, as opposed to outside, the cell (Bókkon et al., 2010). As cells are packed full of potential UPE “detectors” or chromophores, in much closer proximity than examined in long-distance signalling experiments, the prospect of UPE-based signalling seems more feasible. Such proposed “detectors” or “receptors” include aromatic amino acids, such as tryptophan or tyrosine, found in high density in the microtubule cytoskeleton (Craddock et al., 2014). They could also include many other fundamental molecules, from which life evolved as a dissipative structure, usually with double bond structures, and thus range from NADH to DNA, and components of the electron transport chain (Nunn et al., 2020). It could be argued that any biological molecule capable of absorbing a photon will alter the structure/pathway it is in. Recent work by Celardo et al. on super-radiance (a term describing collective emission of a system following a coherent excitation) in microtubules describes the amplification of photon signal by biological structures and may begin to explain how cells may use these initially produced low levels of photons (Celardo et al., 2019). In this manner, the microtubule cytoskeleton, being in close proximity to the mitochondria, a potent source of UPE, may be able to absorb and channel UPE-induced excitation across substantial distances via resonance energy transfer (Kurian et al., 2017). It has even been suggested that the interaction of anaesthetics with these microtubules may be what causes their anaesthetic effect (Craddock et al., 2017; Kalra et al., 2023). The mitochondria themselves, which exist in dynamic, reticulated connected networks, have also been suggested to act as possible “optical waveguides”, facilitating long-distance communication between distant mitochondria. In this manner, microtubule or mitochondrial networks could act as “organic fibre optic nets”, enabling high-speed communication throughout the cell (Thar and Kühl, 2004). In these models, the stacked arrangement of tyrosine and tryptophan residues form aromatic networks (or mitochondria) that allow the propagation of UPE-induced excitation along the length of the cytoskeleton, enables coherent “beats”, analogous to what has been proposed to occur in quantum models of photosynthesis (McFadden and Al-Khalili, 2018). Under such conditions, UPE relay appears a highly likely, fast, and robust intracellular communication mechanism for maintenance of cellular homeostasis.

One salient viewpoint, which we have suggested, is that as cells are full of chromophoric compounds, life may have evolved based on these properties. For example, many components of the electron transport chain, as well as key electron transporters, such as NAD(P)H, can not only absorb photons, but can also re-emit them by fluorescence. From an alternative perspective, cells could be viewed as being full of sunscreen molecules, as these can dissipate excess energy and enable metabolism to occur efficiently (Nunn et al., 2020). It is even possible that photons, and electric fields may have been key in the origins of life–as both can be generated in alkaline thermal vents (Nunn et al., 2022).

### 1.2 Ultraweak photon emission and delayed luminescence

It is vital to make a distinction between two endogenous sources of cellular light–UPE, and a separate photon emitting mechanism known as Delayed Luminescence (DL). In DL, materials, biological or otherwise, exhibit a long-lasting luminescence after the illumination source as been removed or shut off. Like UPE, the intensity of DL is orders of magnitude below typical fluorescence or phosphorescence (Scordino et al., 2014). The distinction we make is the source of excitation that facilitates the emission; in UPE photons are emitted “spontaneously” by metabolic processes (see below), whereas DL occurs following exposure to a light source.

The failure to identify and/or separate the two phenomena is another difficulty the field faces. Many articles describing non-chemical communication effects do not reference or appear to distinguish the two mechanisms, generating uncertainty about the true source of the effect. Moreover, there is evidence to suggest that the intensity of DL, like UPE, is dependent on mitochondrial status (Tian et al., 2022), thus, DL may also be contributing to light-based non-chemical communication. It is therefore essential that future studies correctly account for DL when attempting to study UPE.

## 2 Production and characteristics of UPE

There is compelling evidence that some metabolic reactions within living cells spontaneously produce photons, the intensity of which varies from a few, to several hundred photons per sec/cm^2^, typically with a spectral range of around 200–800 nm (Cifra and Pospíšil, 2014). Fundamentally, UPE occurs during essential metabolic reactions, characterised by molecules moving from high to lower energy states, releasing photons and electronically excited products. Such pathways include the mitochondrial respiration chain, lipid peroxidation, peroxisome and catecholamine biochemistry, as well as oxidation of tyrosine and tryptophan residues in proteins (Bókkon et al., 2010).

The currently accepted mechanism of UPE is that it predominantly originates from reactions of reactive oxygen species (ROS), comprising of reactive molecules and free radicals that are derived from the stepwise reduction of molecular oxygen (O_2_) via high-energy exposure or electron-transfer reactions (Pospíšil et al., 2019). UPE may also arise from the breakdown of reactive nitrile species and the general cessation of electronically excited states (Rahnama et al., 2011; Kobayashi et al., 2014). The nature of ROS and their association with biophoton production is a key consideration. ROS are unavoidably produced by energy generating pathways, including photosynthesis, glycolysis and certain facets of mitochondrial respiration (Rodriguez and Redman, 2005). High levels of ROS are generally toxic due to their formidable capacity to oxidise metabolic products including carbohydrates, proteins, lipids and DNA, whilst excessive ROS build up can eventually lead to a breakdown in normal metabolic function and ultimately cell death (Rodriguez and Redman, 2005). Indeed, elevated levels of ROS characterise “oxidative stress” which underpins a number of age-related conditions, including cancer, Parkinson’s disease and type-2 diabetes (Scialò et al., 2017). However, it is also accepted that the physiological roles of ROS encompass key aspects of signalling and play an important role in the defence against infection, apoptosis, and accelerated ageing (Schieber and Chandel, 2014).

**FIGURE 1:**
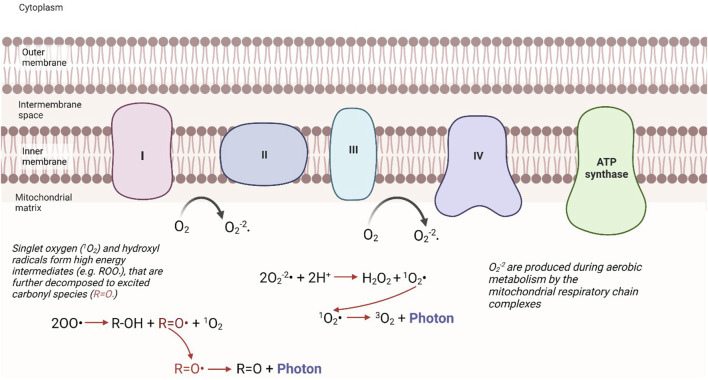
Mechanistic pathways of UPE from the mitochondria. The production of ROS, considered a byproduct of mitochondrial respiration, can lead to the production of singlet oxidation, or excited carbonyl species. When these excited intermediates relax to their ground states, they release this excess energy in the form of a photon. “Modified from Bókkon et al. (2010) (Figure 3)”.

Redox reactions within mitochondria, which generate many reactive, high-energy species, appear to be the central source of UPE in eukaryotic cells; principally from the excited electrons of singlet oxygen and carbonyl groups (Figure 1). Two main pathways appear to exist, firstly the cascade of reactions initiated by complexes of the mitochondrial respiratory chain drive the production of superoxide anions, which in turn leads to the production of singlet oxygen species. Secondly, singlet oxygen and hydroxyl radical species react with nearby biomolecules, such as proteins and lipids, to form high energy intermediates, which upon further decomposition generate excited species such as carbonyl groups (Bókkon et al., 2010). When an excited carbonyl or singlet oxygen returns to a lower energy state, it releases its excess energy as a photon (Figure 1). The photon emission energies of these species is in specific regions of the electromagnetic spectrum: 350–550 nm (blue-green) for excited carbonyl species, 634 nm, 703 nm (red) and 1,270 nm (NIR) for singlet oxygen (Prasad et al., 2018). UPE intensity is proposed to be radically higher inside compared with outside cells (Bókkon et al., 2010), yet under normal conditions the intensity of this emission detected is still extremely low, on the order of tens of photons s^−1^ cm^−1^ (Bókkon et al., 2010). However, as the production of ROS increases with stress, this can be enhanced to hundreds of photons s^−1^ cm^−1^ (Pospíšil et al., 2014).

## 3 Detection of UPE

The greatest challenge in this field is the satisfactory detection and characterization of photons produced by intracellular metabolic processes. As described above, the intensity of such emission is estimated to range from tens to a maximum of one hundred photons s^−1^ cm^−2^ and span the visible to NIR regions of the spectrum. In this section, we will describe in detail the challenges in detecting UPE and suggest the application of certain technologies to navigate these.

A significant consideration associated with the low intensity of cellular emission, is discriminating it from extrinsic light sources including daylight, room light and light from instruments (e.g., indicator LEDs). These can in general be eliminated by having a chamber or box which is completely sealed, as well as performing experiments in rooms with zero light. In short, the experimental chamber needs to be inside another room which is considered pitch black, as is standard across reported UPE detection reported in the literature (Sung et al., 2004; Prasad et al., 2020). However, this is still insufficient to eliminate cosmic rays, radioactive decay and other high energy sources of photons, thus experimental design needs to include management of these artifacts.

The biggest obstacle to detecting UPE is the lack of photons available to measure. Although it is thought the light is in the UV-NIR range and varies with the chemical species that emit it, fully resolved spectra of UPE have never been acquired. To gain spectral resolution, a set up further separates the photon count into discrete bands: with the better resolution, the fewer resultant photons per channel. A 1 nm resolution spectrograph between 300 and 800 nm means 1/500 the signal for each channel or 500 times the detection time for the same signal. Therefore, most reported spectra of UPE have been gathered using a single channel detector and spectral filters, giving a resolution of around 50 nm (Prasad et al., 2020). Each additional optic used in a UPE set up causes further losses of detection, and so adding spectroscopy apparatus will further diminish the signal. For example, a commercially available Czerny-Turner spectrometer (the current instrument of choice for UV-VIS spectroscopy) is specified to have ∼50% loss of signal due to optics coatings without calculating coupling optic efficiencies (Oxford Instruments, 2023). Therefore, further experimental considerations should be taken for increased measurement time.

**FIGURE 2:**
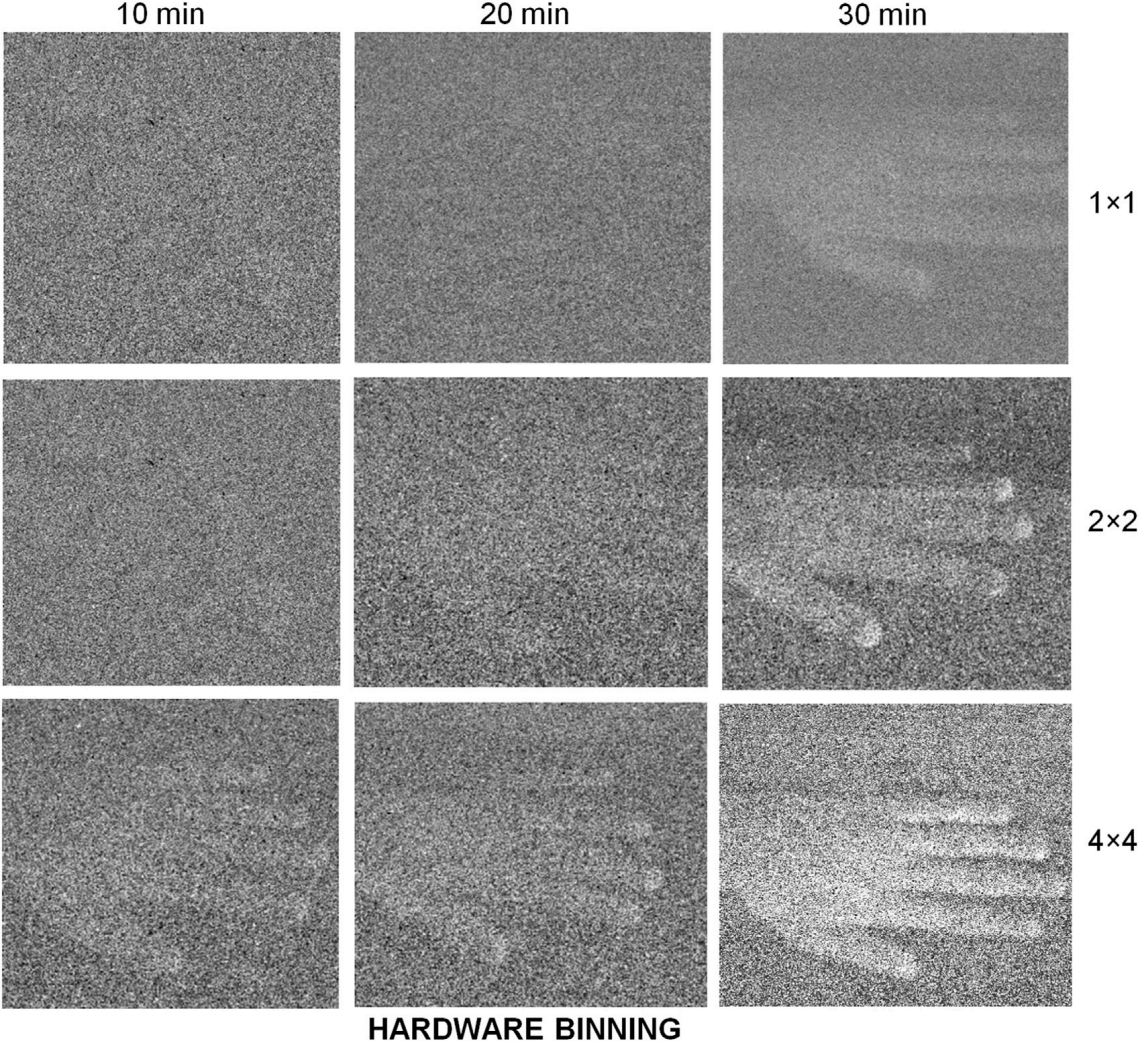
EMCCDs need long exposures to image samples with a very low emission of photons. By binning pixel readout values a lower resolution but higher contrast image can be constructed. Image reproduced from Prasad and Pospíšil (2013) Figure 6B under CC-BY.

Just as obtaining spectra involves dividing the signal into channels in one dimension, attempting to resolve UPE as images, means dividing the photon count between each channel on a 2-dimensional pixel grid, with a lower signal per channel. Also, keeping all the light in focus for a sharp image means sacrificing out-of-focus photons: leading to further losses. UPE imaging studies therefore typically acquire more photons by using larger samples and subjects, with more sources of UPE and longer imaging times. Thirty minutes minimum acquisition is typical and macro-scale subjects are used (the largest being humans on the square meter scale) (Figure 2) (Prasad and Pospíšil, 2013). The experiments reported in these studies, however, do not appear to account fully for DL.

Detectors have a base level of noise and therefore a minimum signal is needed to surpass this background noise. A signal-to-noise ratio of 1 or greater is usually required for adequate data statistics. UPE has been characterised at around 10–100s photons per second per centimetre squared total (Quickenden and Tilbury, 1991), meaning detectors need to have an intrinsic noise level lower than this. Many current photon detectors are described as single-photon detectors by the manufacturers. We have investigated a number of these and found them not ideal for the detection of UPE as expected. Single-photon avalanche diode (SPAD) detectors, for example, are considered ideal for low-light experiments, but since they emit light during detection events (Kurtsiefer et al., 2001), they could greatly interfere with the UPE detection (see specific section below). Electron Multiplying Charge Coupled Device (EMCCD) imaging detectors have single-photon detection sensitivity, but due to multiplication and read-out noise they are not good for quantitatively counting ultra-low-rate photons. It seems some photon-multiplier tubes (PMT) show better promise for low dark count rate (photon counts in the absence of incident light) in digital mode, although their quantum efficiencies (QE, the measure of the effectiveness of a detector to convert photons into electrons), around 20%, are much less than that of single-photon avalanche photodiodes (SPAD) and EMCCD (80%–95%) There are also new scientific complementary metal–oxide–semiconductor (sCMOS) cameras that advertise quantitative single-photon discrimination and may hold the advantage in the coming years. These photon detection systems are explored and discussed in turn further below.

### 3.1 Characteristics of detectors used in UPE detection

#### 3.1.1 Electron Multiplying Charge Coupled Devices (EMCCDs)

An ideal and desirable set up for UPE is one that can resolve the spatial characteristics of the emitted photons. A CCD is a type of light detector wherein an incident photon is converted to an electron and is digitised with an analogue to digital converter (ADC). In an EMCCD, an electron multiplication register is added to improve the signal-to-noise ratio. EMCCDs are rated as having a QE of greater than 90%. Theoretically, these should be an ideal choice for UPE. However, this is not the case due to several limitations in the design electronics of the readout process.

**FIGURE 3:**
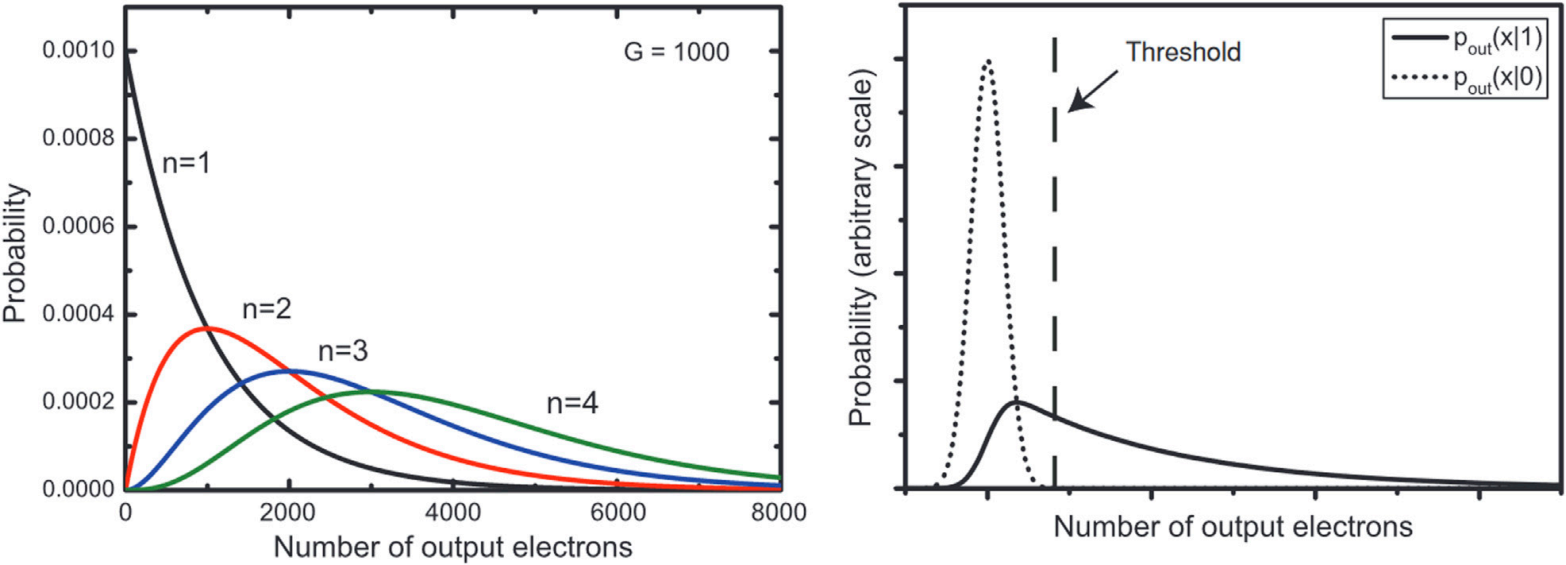
Comparison of possible readings from (left) digit photon readings per pixel and (right) camera background noise *versus* a single photon reading. A threshold applied will either exclude some single photon events or include some thermal events and therefore absolute quantitative readings are not possible from an EMCCD. Image reproduced with permission from (Zhang et al., 2009).

The development of the EMCCD has enabled major advances in biological imaging. It enables a much higher sensitivity, allowing single electrons to be multiplied in number to above noise. EMCCD function by utilising readout wells that are held on a large potential difference where electrons inside can accelerate and liberate an electron from the well material. By repeating this several times, every signal can be increased multiple times. The most detailed images of UPE so far have come from using EMCCDs. However, these can take a minimum of 10s of minutes to hours, so require minimal movement of the sample. A further downside of this method is the lack of quantitative photon counting. It is fair to say that EMCCDs can operate in a “photon counting mode” under certain conditions according to the manufacturers. However, this is not entirely accurate as it is best described as a binary counting method and as such, EM cannot be used as a quantitative counting method. The multiplication is tied to the randomness of the gain process, where there is no definite value, and any particular electron is multiplied up over certain ranges. The range for small numbers of photons overlap with each other and the thermal background to eliminate any exact quantitative assignment (Zhang et al., 2009). Despite this, attempts have been made to use an EMCCD quantitatively by applying calibrated thresholds (Figure 3) (Khaoua et al., 2021).

EMCCDs have been extensively used to detect chemiluminescence, for instance, these include using H_2_O_2_ to initiate a reaction in the biological system (Saeidfirozeh et al., 2018).

Intensified CCDs have also been used for UPE detection. ICCDs use an additional module in front of the CCD which takes in photons and multiplies them using a micro-channel plate. The multiplication process involves a photocathode and a high potential difference (2–4 kV) similar to a PMT, but the output is a phosphor screen coupled to the camera via a lens or fibre bundle. A major drawback is the low quantum efficiency of photon detection at around 30%. CCD images were some of the first high quality images of UPE and showed evidence of an increased photon output due to wounding (Chen et al., 2003).

#### 3.1.2 SCMOS

Another imaging device that can be used for UPE detection are Complementary Metal-Oxide Semiconductor (CMOS) devices. CMOS technology utilises an array of light sensitive pixels to acquire an image, digitizing each signal with separate electronics for each individual pixel. The scientific CMOS (sCMOS) is an improvement to the technology that overcomes the shortcomings of a standard CMOS–offering lower noise, higher speeds, and a greater quantum efficiency (QE), which is now approaching 70% and still improving. However, these quoted values by manufacturers are derived from the different methods of pixel binning. Photons arriving at each pixel have an overlap distribution and low number of photons cannot be distinguished easily. Again, the random arrival of low numbers of photons is not enough to overcome the electronic threshold to register as a signal. Overall, sCMOS is very good at distinguishing signals where 1000s of photons arrive at the same time compared to 15 photons per second as would be expected from UPE. A key technological advantage as well as a weakness of sCMOS is that each pixel has its own read-out circuitry which introduces some variance in low-light level imaging and any electron-multiplication. Repeating this process for every pixel leads to a significant variance and increased background noise. However, currently the single photon level of detection for sCMOS is not yet as good as EMCCDs and therefore are not widely adopted for UPE.

#### 3.1.3 Photomultipliers tubes (PMTs)

Photomultipliers tubes (PMTs) have been used extensively in UPE detection. Unlike CMOS and EMCCDs, these are not imaging devices - unless an x,y scanning method is introduced into the setup (Kobayashi et al., 2006). Early work on detecting UPE used modified Geiger-Mueller tubes. These contain a tube filled with an inert gas which on collision with a photon generates an ionised electron. By holding the gas in a voltage potential the electron is accelerated into more atoms liberating more ionised electrons. This one-to-many generation is then able to be detected by electrical current measurements. The modern evolution of this technology is the PMT. This is a vacuum tube with several curved electrodes held at increasing voltages. The anode has a material that liberates an electron on photon impact. This electron is accelerated into the next electrode where the kinetic impact ejects many electrons. These similarly get accelerated into the next electrodes until millions of electrons are read out as a current at the cathode. A guide to technical aspects of PMTs can be found online (Photonis, 2002).

**FIGURE 4:**
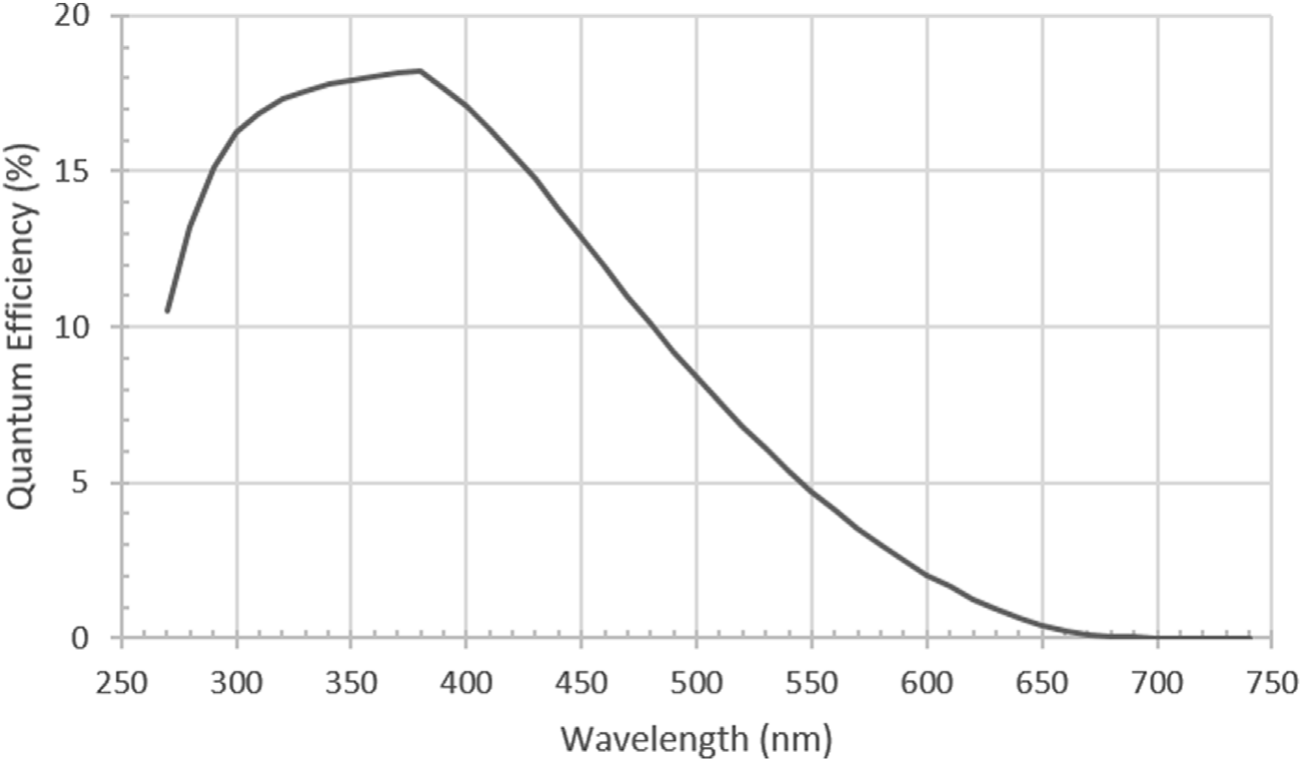
Typical Quantum Efficiency (QE) of a low dark count rate PMT [H11870-01Hamamatsu Photonics, Japan] Reproduced with permission from Ref (Mackenzie et al., 2024) Figure SI-4.

PMTs typically have a quantum efficiency of 20% or less, considered low when compared to EMCCDs and sCMOS. However, PMT technology has been refined to be able to give dark count rates of <50 per second and able to reach single digits dark count rates with cooling. They also have the advantage of giving real-time outputs and so can be used to adjust and optimise detection apparatus setups. Recent coupling with photon-counting hardware enables better protection against cosmic rays contaminating signals with large spikes. All this combines to give PMTs a great signal to noise ratio at the 40–100 photon per second detection rate needed for UPE detection. It is also worth noting that PMTs are normally rated as relative efficiency and a conversion to quantum efficiency is needed. So that higher photon energy (<400 nm) on the PMT is more likely to generate a higher current than a lower energy photon (>600 nm). On photon counting devices this is factory calibrated to enable easier quantum efficiency conversions (Figure 4).

#### 3.1.4 Single-photon avalanche diodes (SPADs)

A promising new technology for UPE detection is the development of single-photon avalanche diodes. These use a silicon diode detector negatively biased into the avalanche breakdown region. This means that a single photon can push the semiconductor into the conducting region and pass a current backwards through the diode. However quenching circuitry will stop the current on detection and re-bias the diode ready to detect again all within ∼40 ns? Since the QEs of SPADs can reach 80% at some wavelengths (currently in the red to infra-red), this seems to be a direct upgrade for some PMTs. However, the avalanche process itself is known to emit light (Kurtsiefer et al., 2001). This may contaminate the dark chamber and interact with the biological sample producing spurious results. Therefore, they have so far been seldom used in UPE work.

### 3.2 Detection summary

The main method for UPE detection, PMTs, are still the best way of getting quantitative counts of photons from samples. However, with the development of electron-multiplying technology we can sacrifice precise photon counts for imaging signal above the noise level and many new camera technologies are achieving this. Careful choice of scientific question and experimental design are the most essential when trying to measure UPE.

## 4 Applications of UPE and DL

Whilst the full detection and characterisation of the phenomena still evades researchers, emission of light from cells that is dependent on its metabolic status, whether it is DL or UPE, provide potential, non-invasive markers for assessing a variety of physiological functions. This approach has been applied to a broad range of subjects, with recent studies in plant biology, food quality, environment, pollutants and drug efficacy all employing UPE/DL detection as a novel means of enquiry. In the sections below, we briefly run through work describing potential applications of spontaneous light emission from cells.

### 4.1 UPE in plant biology and agriculture

Many investigations now exploit the direct relationship between stress, ROS production, and UPE/DL to provide a quick, non-invasive diagnostic tool for assessing plant physiology (Komatsu et al., 2014). Various stimuli have been shown to alter UPE/DL in plants; Prasad et al. demonstrated that mechanical wounding to discrete regions of the *Arabidopsis* plant induced high levels of UPE, a phenomena that lasted for several hours and was strictly limited to injured areas (Prasad et al., 2020). UPE has also been used to track red bean plant and root function following exposure to the stressors of elevated salt concentration and drought (Ohya et al., 2000; Ohya et al., 2002). Additionally, these studies revealed that the degree of UPE was altered, not only by the intensity of the insult, but was dependent on the developmental stage of the plant. Studies on mung beans showed an increasing signal as the germinating beans grew larger and our own work found a rapid increase of emission during growth of secondary roots (Rafieiolhosseini et al., 2016; Gallep and Robert, 2020; Mackenzie et al., 2024).

Indeed, UPE is tightly coupled to the physiological state of a plant, and changes significantly in response to a variety of biotic stressors, including pathogenic elicitors such as microorganisms (fungi, bacteria, viruses) and microbial cell components (Kato et al., 2014). Proposed advances in agricultural techniques rely on the so-called “elicitor-responsive photon emission” effect to detect and identify a given threat by interpreting a plant’s characteristic UPE response (Bennett et al., 2005). A key component of a plant’s defence is the hypersensitive response, which involves activation of the host resistant gene (R gene) at an infection site, resulting in localised cell death to eliminate invading pathogens. R gene initiated activation of this signal cascade leads to ROS production and associated biophoton release, a response observed in a variety of different plant hosts (Kobayashi et al., 2006). For example, Duan et al. demonstrated that UPE can distinguish between normal and infected wheat (Duan et al., 2019).

Studies have also suggested that a cultured seed’s UPE profile could be used to identify two essential components to agricultural management, namely, germinative ability (potentiality of growth) and vigour (post-germination performance potential). The intensity of UPE observed in viable soybean seeds is double that of non-viable seeds (Veselova et al., 1985). Furthermore, germinative ability decreases with storage time, indicating delayed luminescence could be monitored to identify viable air-dry stored seeds (Zeiger, 1998). Interestingly, biophoton signalling has been hypothesized to function as a key transducer for optimal seed growth; the attenuation of light underground might enable a plant’s roots to exploit UPE for relaying key environmental feedback on soil humidity, temperature, and oxygen. In turn, this informs on seasonal patterns and would enable the plant to select the most favourable window for germination (Footitt et al., 2016).

### 4.2 Applications of UPE in food quality, environment and pollutants

The use of UPE in food quality research was pioneered by F.Popp, who demonstrated that it was possible to distinguish between conventionally grown and supermarket tomatoes (Popp et al., 1988). It has also been shown that UPE may be used to assess the quality of organic eggs (Grashorn and Egerer, 2007). Further studies exploiting the after-glow of *delayed luminescence* have been used to investigate a varied assortment of agricultural themes, including the relative ripeness of tomatoes, the intensity and type of fertiliser, and the effects of different farming styles on milk composition (Musumeci et al., 2003)^,^ (Stolz et al., 2019).

Activation of a plant’s stress pathways and the associated increase in UPE can reflect not only physiological insult, but also a response to external environmental stimuli. This can manifest from human-borne pollutants such as insecticides and other chemical agents, or stressors such as UVA radiation (Lambing, 1992; Rastogi and Pospíšil, 2013). Highly sensitive imaging techniques have been used to investigate the effects of UVA on *Arabidopsis thaliana* plants, with the resulting UPE profiles showing distinctive decay patterns in specific regions of the visible spectrum (Rastogi and Pospíšil, 2013). This response is not limited to plants, with additional organisms reacting in a comparable manner to environmental stressors; for example, lipid peroxidation has been shown to increase photon emission in the algae *Chlamydomonas reinhardtii* (Prasad and Pospíšil, 2011).

### 4.3 Applications of UPE in disease and drug development

The link between ROS production and UPE has not been limited to plants, affording a potentially non-invasive means of investigating oxidative stress, immune response and drug efficacy in humans (Kobayashi et al., 2014). Sustained exposure to high levels of ROS, via elevated production or the failure of antioxidant defences, is associated with the pathophysiology of multiple diseases (Kobayashi et al., 2014). Furthermore, the failure to produce ROS effectively in response to stressors is also indicative of metabolic dysfunction (Burgos et al., 2017). The intrinsic link between ROS production and biophoton release means that monitoring UPE in living organisms represents a real-time, non-invasive method of early-state disease diagnosis (Ives et al., 2014).

Neutrophil granulocytes, comprising around 40%–70% of all white blood cells, are an essential frontline component of the innate immune system. A key aspect of their function is the cell’s respiratory burst, which manufactures high levels of ROS species to destroy invading pathogens. This increase in oxidative stress and ROS, observed in disorders such as Parkinson’s disease, atherosclerosis and cancer, is a key target for pharmacological intervention (Scialò et al., 2017)^,^ (Burgos et al., 2017). Importantly, the UPE accompanying this phenomenon has been demonstrated in several conditions; including acute myeloid leukaemia (Burgos et al., 2018), cardiovascular disease (Rizzo et al., 2016), and a mouse model of rheumatoid arthritis (van Wijk et al., 2013). Furthermore, antioxidant drugs that inhibit enzymes such as NADPH oxidase and myeloperoxidase, have been shown to demonstrate dose-dependent effects on UPE (Burgos et al., 2018). Monitoring UPE therefore represents a future low-cost, label-free tool to monitor pharmacological intervention and drug therapies for the wide range of diseases comprising a ROS response.

Indeed, ROS are also involved in ageing of the skin and the pathogenesis of a number of allergic and inflammatory skin diseases (Okayama, 2005). Thus, one of the more promising applications of UPE in diagnosis is within the field of dermatology: skin provides a convenient *in vivo* target, offering high accuracy of clinical results, as diagnostic tools do not have to interfere with other tissues before reaching the target (Ou-Yang, 2014). UPE has been proposed as a non-invasive skin diagnostic technique since 1997, where Cohen and Popp showed that photon emission changes with biological rhythm and can reflect the left-right symmetry of the body, indicating that UPE is associated with human physiology (Cohen and Popp, 2003). Later, other experimental research used PMTs and coupled camera to observe alterations of ultra low photon emission of the human hand skin, showing that the UPE count doubled when hydrogen peroxide was topically applied on skin, indicating that photon emission from the hand associated with skin physiology (Rastogi and Pospíšil, 2013).

Quantitative UPE analysis is already being used to test antioxidant therapies, including the efficacy of sunscreens and dermal-applied therapeutic agents for acne and dermatitis (Ou-Yang, 2014). Moreover, several *in vivo* studies have reported significantly decreased UPE in skin treated with antioxidants such as β-carotene (Sauermann et al., 1999), α-glucosylrutin (Hagens et al., 2008) glutathione and coenzyme Q10 (Rastogi and Pospíšil, 2011). Here, UPE detection represents an improvement over previous techniques, such as skin biopsies, as it provides a greater degree of non-invasiveness, a longer window of assessment post-stimulus and avoids the use of experimental animals or exposure to ROS for accurate monitoring (Ou-Yang, 2014). UPE detection is also being incorporated into the evaluation of novel therapies such as low-intensity light therapy (LILT), a fast-growing technology used in the treatment of conditions such as psoriasis, that require stimulation of healing, pain relief and restoration of function (Tafur et al., 2010). A recent study by Esmaeilpour and colleagues also used UPE to examine the differentiation of neural stem cells in conjunction with nanoparticle delivery (Esmaeilpour et al., 2020), indicting UPE detection could be used in conjunction with emerging adjuvant and primer technologies across a range of disciplines.

### 4.4 Applications of UPE in brain function

The role of UPE in the brain has attracted particular attention for its potential to address some of the “unsolved problems in neuroscience” (Kumar et al., 2016). UPE has been detected in various brain tissues, including rat hippocampal slice and rat cerebellar granule neurons. In both, the level of weak bio-chemiluminescence was also found to increase upon potassium or calcium induced-depolarization, and decrease in response to treatment with the sodium channel-blocker tetrodoxin, suggesting a correlation between biophoton intensity and neural-metabolic activity, in turn is postulated as a new mechanism for neural information processing (Tang and Dai, 2014; Esmaeilpour et al., 2020). UPE activity in the brain has been theorized to have far-reaching consequences, ranging from the mechanism behind the phenomenon of retinal dark noise, to explanations of consciousness. In such explanations, interactions between UPE and microtubules are considered to be central. The arrangement of aromatic tubulin dimers which make up microtubules facilitate the diffusion of photo-excitation induced electronic energy over considerable distance, an effect which reduced in the presence of anaesthetics (Kalra et al., 2023), and in fact is modulated by the potency of the anaesthetic tested (Craddock et al., 2017). Further, the arrangement of microtubules have been simulated to enable non-trivial quantum effects that are resistant to decoherence (Hameroff et al., 2002). In this way are UPE, microtubules, and quantum biology are theorized to contribute or give rise to conscience (Craddock et al., 2016), perhaps most predominantly in the Orchestrated Objected Reduction (Orch OR) theory (Hameroff and Penrose, 2014).

### 4.5 Summary of applications

Whilst the applications of UPE does hold promise, there remains limitations and challenges that need to be addressed. One difficulty that underpins many of these applications, and indeed UPE research as a whole, is the extremely low numbers of photons being generated. This presents a challenge in dermatological applications, for example, as the skin has layered structure with different optical properties between layers. This is exacerbated by the presence of pigments in the skin, such as melanin and haemoglobin that can further obfuscate a UPE signal (Ou-Yang, 2014). Furthermore, whilst many papers tout the predicted benefits of UPE as a diagnostic tool, there are currently no trials that seem to demonstrate that UPE detection has a significant advantage over existing diagnostic techniques.

## 5 Discussion/conclusion

From the first experiments of Alexander Gurwitsch in the 1920s, the existence of photons from biological activity–mitogenic radiation, metabolic photons, biological autoluminescence, biophotons, ultraweak photon emission, or any other of its monikers, are now becoming more established. Yet their role within an organism remains under researched - despite the results of such work having enormous consequences on models of cellular signalling and having wide-ranging and important applications in agriculture and medicine. As we have suggested, the existence of essential chromophoric molecules that life is built upon, and the possibility that light, either from the sun, or from an alkaline thermal vent, could have played a role in its origins, is perhaps, is also intellectually suggestive. That quantum mechanics is playing more of a role in biology that previously thought could also be indicative (Marais et al., 2018). There is still a question over what precisely generates UPE, although ROS has been identified as a key source. As we suggested, there is also a fundamental reason why UPE detection requires external manipulation to produce enough photons to be detected via a ROS process; the evolution of sunscreens would have been essential for early life. However, the underlying physics still applies: the movement of electrons, and thus the potential for excited states, is what defines biology, which of course means the potential for the generation of ROS and photons–this is actually embraced by the field of chemiexcitation. This is also tightly linked to adaptive thermodynamics and the description of life as a far from equilibrium, self-organising structure that has emerged from an energy potential to dissipate energy or direct it to where it is most useful, and possibly explain ageing, and the flipside roles of hormesis and inflammation (Thar and Kühl, 2004).

Some fundamental questions remain, including: what is the role of the photons produced in biology? Are they simply a by-product of reactions in biosystems or do they have a role in biological health and intra/intercellular communications? Although ROS has been identified as a key source of detectable emission, what are the “detectors” and “inductors” of the communicative effects? Moreover, apart from UPE that are difficult to detect, DL photons are also abundant, some of which are not exclusive to biological samples. The combined role and effect of DL and UPE that are produced after the complete decay of DL is still unknown. This leads us to wonder whether UPE is part of a fundamental homeostatic system in biology.

The slow uptake of research into UPE is primarily due to the difficulties in the experimentation. Understanding the properties of the detector used is imperative, as well as designing an experimental environment where ambient light is eliminated. A fully light impregnable chamber would still not eliminate intrinsic sources of light. Radioactive decay of atoms within the sample will cause detectable photon emission events, but generally using small samples at a level below signal detection minimises this background noise. Any stimulated photon effect (fluorescence/phosphorescence) will usually be at a level much higher than that of natural UPE (by several orders of magnitude). Similarly, inducing chemical reactions can cause photon emission at levels not detectable to human eye, but registered by the detectors. Since the source of UPE may be a product of a reaction (e.g., decay of generated OH radicals) then any induced chemical generation effect may show a similar detection profile while not actually activating the biological pathway under study. Increased UPE has also been shown to occur with increased temperature (Kobayashi et al., 2014), however detector background counts also increase with increased temperature, so cooling and isolation of detectors need to be implemented in all UPE studies. When measuring UPE, the initial time period (from minutes up to 24 h) is dominated by DL if not carefully avoided. It should also be noted that any exposure of the detectors to strong light will show an initial increase in signal as generated excited states in the detector material decay and emit further light, as well as cause possible damage to the detector. Many long-term studies reported in the literature, especially imaging studies, may very well be observing mostly DL rather than UPE effect. The contribution of DL in UPE studies should be carefully considered. All these factors have to be carefully balanced to get meaningful information from UPE studies in a timely manner.

UPE research thus presents a field of great technical challenges and long history but offers a further insight into the function of ROS, the inner workings of the cell and an understanding of qualitative difference in living entities.
